# Multicenter evaluation of Fourier transform infrared (FTIR) spectroscopy as a first-line typing tool for carbapenemase-producing *Klebsiella pneumoniae* in clinical settings

**DOI:** 10.1128/jcm.01122-24

**Published:** 2024-11-27

**Authors:** Ana Candela, David Rodríguez-Temporal, Pilar Lumbreras, Paula Guijarro-Sánchez, Manuel J. Arroyo, Fernando Vázquez, Alejandro Beceiro, Germán Bou, Patricia Muñoz, Marina Oviaño, Javier Fernández, Belén Rodríguez-Sánchez

**Affiliations:** 1 Servicio de Microbiología, Complexo Hospitalario Universitario A Coruña, Institute of Biomedical Research A Coruña (INIBIC) 571757 , A Coruña, Spain; 2 Servicio de Microbiología y Enfermedades Infecciosas, Institute of Health Research Gregorio Marañón (IiSGM), Hospital General Universitario Gregorio Marañón 16483 , Madrid, Spain; 3 Grupo de Microbiología Traslacional, Instituto de Investigación Sanitaria del Principado de Asturias 594910 , Oviedo, Spain; 4 Servicio de Microbiología, Hospital Universitario Central de Asturias 16474 , Oviedo, Spain; 5 Clover Bioanalytical Software, Granada, Spain; 6 CIBER de Enfermedades Infecciosas (Instituto de Salud Carlos III, CIBERINFEC, CB21/13/00055) 637284 , Madrid, Spain; 7 CIBER de Enfermedades Respiratorias (Instituto de Salud Carlos III, CIBERES CB06/06/0058) 568067 , Madrid, Spain; NorthShore University HealthSystem Department of Pathology and Laboratory Medicine, Evanston, Illinois, USA

**Keywords:** Fourier transform infrared spectroscopy, *Klebsiella pneumoniae*, bacterial typing

## Abstract

Early use of infection control methods is critical for preventing the spread of antimicrobial resistance. Whole-genome sequencing (WGS) is considered the gold standard for investigating outbreaks; however, the turnaround time is usually too long for clinical decision-making and the method is also costly. The aim of this study was to evaluate the performance of Fourier transform infrared (FTIR) and artificial intelligence tools as a first-line typing tool for typing carbapenemase-producing *Klebsiella pneumoniae* (CPK) in the hospital setting. For this purpose, we analyzed 365 CPK isolates from two tertiary hospitals in Spain in parallel by applying unsupervised principal component analysis (PCA) and supervised algorithms (artificial neural network [ANN], support vector machine [SVM] linear, SVM radial basis function in the IR Biotyper software, and random forest in the Clover MSDAS software). Concordance with FTIR clustering considering the sequence type (ST) and the clonal cluster, obtained by cgMLST for reference purposes, was measured using the adjusted Wallace index (AWI), yielding values of 0.611 and 0.652, respectively. Different regions of the spectra were studied in relation to repeatability and reproducibility, and the polysaccharides region proved the best for FTIR differential analysis. The best results for accuracy were obtained using the ANN algorithm in the IR Biotyper software, with 80.5% of correct prediction. Regarding accuracy, the poorest results were obtained for isolates belonging to ST392 (55.5%) and the best results for ST307 (94.4%). The findings demonstrate the utility of the FTIR method as a rapid, inexpensive, first-line typing tool for detecting CPK, preserving WGS for confirmation and further characterization.

## INTRODUCTION

Nosocomial infections represent serious threats to hospitalized patients and are also of major concern in public health systems because of the clinical impact and associated costs (1). In addition, novel pathogens and antimicrobial resistance are increasingly identified owing to advances in medicine and especially in critical care. Rapid and reliable typing of bacterial isolates is essential for detecting possible transmission routes of pathogens in the hospital setting, as well as for identifying bacterial reservoirs and for preventing and evaluating infection control efforts.

*Klebsiella pneumoniae* is a frequent cause of nosocomial outbreaks and warrants immediate infection control measures (2). *K. pneumoniae* also has a high capacity to acquire resistance mechanisms and become multidrug resistant, and identifying and typing are therefore also important in order to prevent and contain spread of this pathogen (3). Special attention must be given to carbapenemase-producing *K. pneumoniae*, a combination of resistance mechanism and bacterium that is very challenging to treat and control (4). Whole-genome sequencing (WGS) has emerged as the gold-standard typing method, especially after the SARS-CoV-2 pandemic, but it is yet a costly procedure, not available for routine diagnosis in many clinical laboratories from primary and secondary hospitals (5, 6). Moreover, these techniques are time consuming, with turnaround times ranging from days to weeks. They also tend to be used retrospectively, after the outbreak has been epidemiologically identified, and not for real-time typing of strains in routine microbiology laboratories.

Fourier transform infrared (FTIR) spectroscopy is based on the absorption and transmission of light in the infrared region of the electromagnetic spectrum, converting it into a spectrum after mathematical (Fourier) transformation. This absorption depends on the nature of the chemical bonds present in a sample and produces different patterns. These patterns vary according to the functional groups present in biomolecules such as carbohydrates, lipids, and proteins. The underlying assumption of the technique is that related strains will have a similar composition and thus more congruent FTIR spectra. Comparison of characteristic spectra or “molecular fingerprint” can thus be used to identify organic samples, such as bacteria, and even for typing purposes (7–9). The method represents a rapid alternative to conventional typing methods, providing results in less than 3 hours and thus making real-time typing feasible in most clinical microbiology laboratories. Identification of the similarity between transformed spectra can be improved by using different analytical models based on artificial intelligence (AI) algorithm tools such as machine learning. It is thus important to evaluate how these algorithms can help in the identification of certain patterns in bacteria to recognize possible outbreaks. Furthermore, the need for between-center comparison for decision-making in infection control leads to reproducibility being a matter of great concern.

The overall aim of this study was to evaluate the discriminatory power of FTIR spectroscopy as a first-line typing tool for carbapenemase-producing *K. pneumoniae* in the clinical microbiology laboratory, relative to WGS analysis. The study also evaluated the performance of the analysis conducted with the basic features included in the IR Biotyper software (Bruker Daltonik, Germany) in comparison with the discriminatory power of different AI algorithms implemented in the Clover MS Data Analysis Software (Clover MSDAS) platform (Clover Biosoft, Spain). Finally, the reproducibility of the model technique was evaluated by performing the same study in two centers.

## MATERIALS AND METHODS

### Bacterial isolates

**Fig 1:**
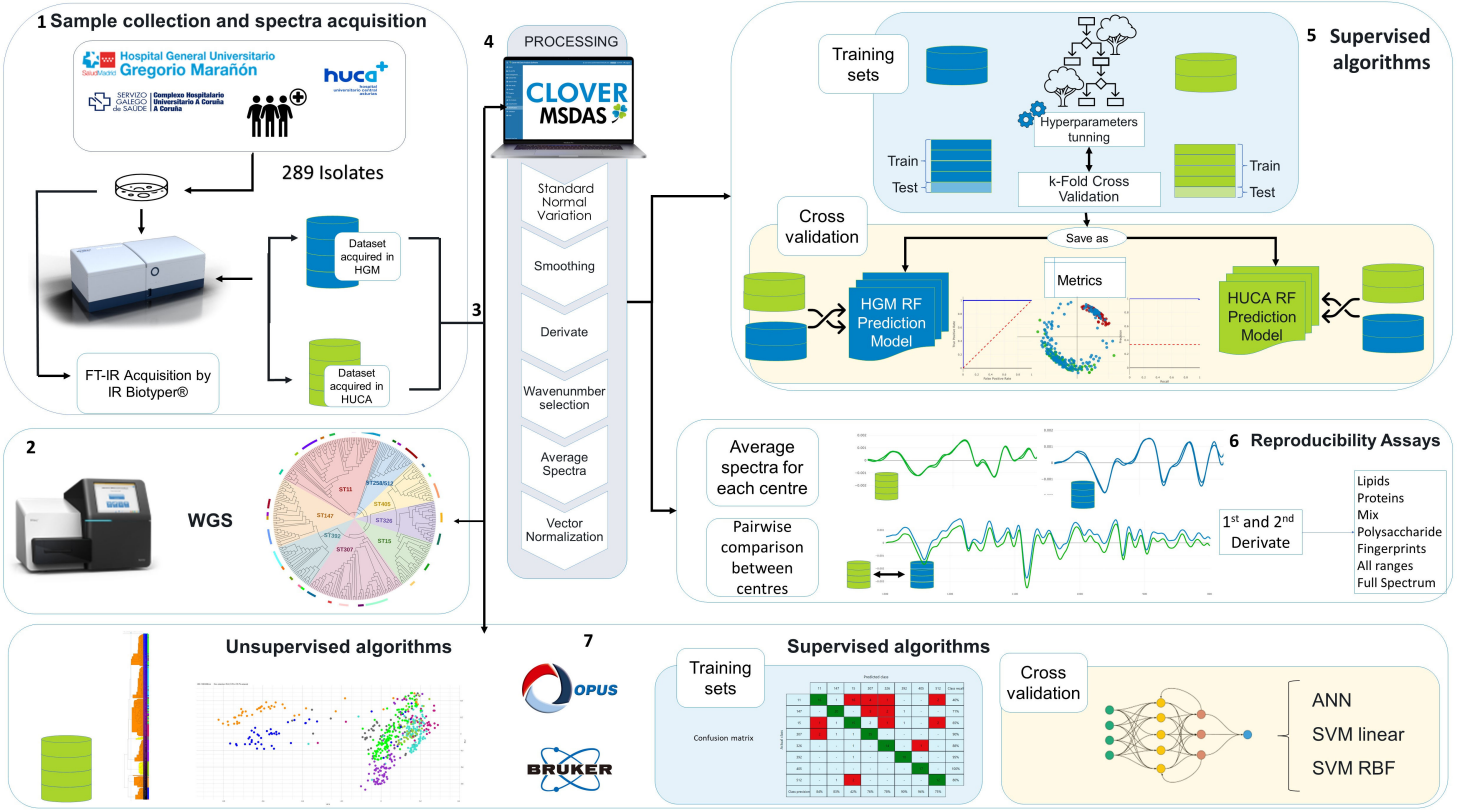
Workflow for the evaluation of FTIR spectroscopy for carbapenemase-producing *K. pneumoniae* typing. (1) Sample collection and spectra acquisition: samples were isolated from infected patients; pathogens were cultured and further characterized by FTIR. All isolates were analyzed in parallel in two tertiary hospitals, Hospital General Gregorio Marañón (HGM) and HUCA. (2) WGS: all isolates were genotypically characterized by WGS and the epidemiological relationships between the isolates established. (3) Analysis: isolates were analyzed using IR Biotyper software (supervised and unsupervised algorithms) and Clover MSDAS software (supervised algorithms and reproducibility studies). (4) Preprocessing in the Clover MSDAS software: the mass spectra profiles were extracted from the IR Biotyper and preprocessed with Clover MSDS by applying smoothing, derivate, wavenumber selection, obtaining average spectra, and finally vector normalization. (5) Processing in the Clover MSDAS software: data were split into training and validation sets. Data were labeled with the information regarding the sequence type (ST), the cluster obtained by cgMLST, and the location of origin. A peak matrix was generated and used as input data to the random forest (RF) supervised machine learning algorithm. The algorithms were trained, and the hyperparameters were optimized. The training step was then evaluated by calculating the resulting metrics from a *k*-fold cross validation method. The metrics reported for validation are accuracy and the area under receiver operating characteristic curves (AUROC). (6) Repeatability and reproducibility: studies were conducted for the same isolates in both centers at different wavelength regions for FTIR discrimination. (7) Processing in the IR Biotyper software: unsupervised and supervised algorithms were evaluated. Regarding unsupervised algorithms, the adjusted Wallace index (AWI) was calculated for concordance between WGS and FTIR. For supervised algorithms, three different algorithms were evaluated: artificial neural network (ANN), support vector machine (SVM) linear, and SVM radial basis function kernel (RBF). Results for accuracy were obtained.

The study included a representative collection of 365 unduplicated carbapenemase-producing *K. pneumoniae* clinical isolates (see Table S1). Among the 365 CPK studied, 289 belong to the Complejo Hospitalario Universitario A Coruña (CHUAC) and were collected during a nationwide survey of carbapenemase-producing Enterobacterales involving 23 hospitals throughout Spain. The survey was promoted by the Spanish Society of Infectious Diseases and Clinical Microbiology and by the Spanish Network for Research in Infectious Diseases. The BioProject accession numbers for the strain genomes are PRJEB39112 and PRJEB42440 (10). The other 76 isolates are part of a collection owned by the Hospital Universitario Central de Asturias (HUCA). The BioProject accession number for the strain genomes is PRJNA718833 (11). All isolates were identified by matrix-assisted laser desorption ionization time-of-flight mass spectrometry with MALDI Biotyper Smart (Bruker Daltonics, Bremen, Germany). The isolates were screened for carbapenemase production according to the screening cut-off values recommended by the European Committee on Antimicrobial Susceptibility Testing (EUCAST v14.0), i.e., with meropenem or ertapenem MICs higher than 0.125 mg/L. Identification was later confirmed by genome sequencing along with confirmation of the resistance mechanisms. All isolates were characterized by WGS and FTIR spectroscopy according to the workflow outlined in Fig. 1.

### Genome sequencing and analysis

All isolates were characterized by WGS. The 289 isolates from the CHUAC collection were sequenced in parallel using short-read (Illumina MiSeq benchtop, Illumina) and long-read (MinION, Oxford Nanopore Technologies) approaches. The other 76 isolates, from the HUCA collection, were sequenced using Illumina technology. Resultant long and short reads from each isolate were assembled together using the Unicycler v0.4.6 hybrid assembler. The assemblies obtained were finally annotated using Prokka v1.13 (12). .

The total antimicrobial resistance gene content of the isolates was analyzed *in silico* using ResFinder v3.2 (13) software, ARMFinderPlus (14), and the Comprehensive Antibiotic Resistance Database (13). The resistance genes were annotated with the information obtained with the different tools. ABRicate with the NCBI database was employed to identify contigs carrying carbapenemase genes (15), using minimum coverage and identity thresholds of 80%. Plasmid replicon analysis isolated contigs were analyzed using MOB-suite with default parameters to identify and characterize plasmid replicons (16), focusing on replicase types. This step assessed the potential association of carbapenemase genes with plasmid structures; then, MOB-suite results were associated with the corresponding sequence type (ST) of each isolate.

To determine the relationships between various STs and the clonal relatedness in our collection, the following analysis was conducted. The genomes were annotated with BAkta (17), and the annotated genomes were then processed with Panaroo to create a core genome alignment (18); both tools were executed using default settings. The core genome alignment was then used as input for RAxML in which the general time-reversible model and a gamma correction for site rate variation were applied (19). Bootstrapping was inferred for up to 1,000 replicate trees, and the different starting trees converged to one unique topology before 500 replicates. The tree was generated using the interactive tree of life online tool (20).

Clonal relatedness was established using the aforementioned methodology. Those isolates with a cgMLST cut-off of ≤18 single nucleotide polymorphisms (SNPs) and originating from the same monophyletic group as determined by RAxML were deemed highly clonally related (21).

Since the default settings used by FTIR analyze the region of the polysaccharides, we tried to elucidate the potential impact of the polysaccharide-associated genes in the accessory genome. The accessory genome was defined as genes present in less than 95% of the analyzed strains. An in-house script was used to extract the accessory genes from the pangenome previously obtained by using Panaroo (18). The protein domains associated with polysaccharide biosynthesis and modification were identified by searching the Pfam database (22). Fourteen relevant domains were identified: PF00534, PF00535, PF02706, PF04932, PF02397, PF01943, PF02719, PF03808, PF04101, PF13440, PF13704, PF14667, PF03935, and PF01075. Seed alignments for each domain were downloaded in Stockholm format, and hidden Markov models specific to each domain were built using these alignments. To calculate intra-ST diversity, pairwise Jaccard distances between polysaccharide-related gene profiles were computed for each pair of genomes within each ST. The average intra-ST diversity was obtained by calculating the mean of these distances for each ST. Statistical significance of the differences in intra-ST diversity among STs was assessed using a t-test (α = 0.05). The t-test was performed using the SciPy library in Python (version 3.8).

### FTIR spectrum acquisition

Prior to FTIR analysis, strains were cultured at 37°C in Columbia sheep blood agar for 24 h under aerobic conditions. A 1-µL loop of the bacterial biomass was suspended in 50 µL of sterile water and homogenized with a metal rod and vortexed. Fifty microliters of 70% ethanol was added to the suspension, which was then vortexed, and 15 µL of the mixture was spotted on the FTIR silica plate (23). FTIR spectra were obtained using the Bruker IR Biotyper (Bruker Daltonics) following the manufacturer’s instructions. Isolates were analyzed in triplicate along with two standards (Bruker Infrared Test Standard 1 and 2) as recommended by the manufacturer. Measurements that did not meet the default quality criteria (0.4 < absorption < 2; signal-to-noise ratio > 40; fringes [×10^−6^], <100) were excluded from further analysis. All samples were analyzed in parallel in the HUCA and in the Hospital General Gregorio Marañón (HGM), Spain.

### FTIR spectrum analysis

#### Unsupervised models

The 365 isolates were analyzed using Biotyper software (OPUS, Bruker Daltonik, Germany) following the manufacturer’s instructions. Briefly, spectra of all samples were acquired in triplicate with default settings (wavelength region 800 cm^−1^ to 1,300 cm^−1^ or the polysaccharide region). Only isolates that yielded at least two out of three satisfactory readings in both centers were considered further, in this case 228 *K*. *pneumoniae* from the CHUAC collection and 61 from the HUCA collection (Table S1), including a total of 289 isolates. After vector normalization, the average spectra for each isolate were constructed on the basis of their respective replicates and processed automatically with the IR Biotyper software, by applying the second derivative. Samples that did not meet the quality criteria were excluded from further analysis. The similarity of the spectra was determined by generating a dendogram based on hierarchical cluster analysis and principal component analysis (PCA) and using the Euclidean metric distance and the average linkage method. This linkage type has the advantage of being independent from the number of isolates to compare, enabling study of totally different data sets and defining cut-off values for isolates that are undistinguishable, closely related or unrelated. The FTIR software automatically suggests a cut-off value for clustering based on Simpson’s diversity index, with lower cut-off values leading to definition of more clusters. FTIR spectra of two or more isolates separated by a distance less than or equal to the designated cut-off value were considered to belong to the same FTIR cluster. Use of metadata is possible, and the cut-off value can then be recalculated under the new criteria. To evaluate the classification according to a particular quality criterion, the software calculates a coherence value: the cophenetic correlation coefficient. This coefficient ranges from 0 to 1, where 0 indicates that all isolates from the same cluster are different, and 1 indicates that all isolates from the cluster are identical. In the present case, two different criteria were considered to generate the dendogram: the ST of the isolate and the genome sequencing cluster obtained by WGS.

#### Clustering concordance

The adjusted Wallace index (AWI) (24) was used to assess the concordance of the clustering results obtained by FTIR relative to WGS, considered the reference method. The AWI is a standard metric commonly used in cluster validation research and compares the agreement of two methods where one is considered the reference method. Values close to 0 signify random occurrence, while those close to 1 indicate that the cluster assignments closely resemble each other. The AWI concordance was determined using mri v1.0.1. All analyses and all packages were performed using R Statistical Software (v4.1.2; R Core Team 2021) and downloaded from the CRAN repository. The AWI was then used to compare the level of agreement between FTIR and WGS clonal clustering but also between FTIR and ST classification obtained by WGS.

#### Supervised models

For use of supervised models, the isolates were randomly divided into training and validation sets, which included 197 and 92 isolates, respectively. Using the IR Biotyper software, the training data set was used to build a predictive model based on artificial neural network (ANN), support vector machine (SVM) linear, and SVM Radial Basis Function kernel (RBF), by using a *k*-fold cross validation method (*k* = 4). Supervised models were constructed using the second derivative and the polysaccharide region. The gold standard for comparing the results of FTIR was the clonal relationship of the isolates expressed by the ST identified during sequencing of the isolates. The results of the FTIR validation were expressed with scores, with the following intervals: 0.001–0.999, indicating a reliable result; 1.000–1.999, indicating low reliability; and ≥2.000, indicating an unreliable result. The outlier scores ranged between 0 and 10. The scores describe how close the sample is in relation to the training set and are expressed as a type of z-score (according to Fisher’s distribution). A value of 0 indicates that the sample is probably part of the training set. Values up to 1 and 2 are well within the expected range for members of the trained class. Larger values indicate novel events (or outliers), i.e., they probably do not belong to the classes in question. The same set of training and validation isolates was evaluated in parallel in the HGM and HUCA centers and was subjected to cross validation.

In the Clover MSDAS software, the raw spectra were exported from the IR Biotyper and processed as follows: standard normal variate transformation, smoothing using a Savitzky-Golay filter (window length: 9, polynomial order: 3), and vector normalization in all spectra after second derivation. The absorbance bands of the spectra were filtered by selecting different regions such as the membrane lipid region (from 3,000 to 2,800 cm^−1^), protein region (from 1,800 to 1,500 cm^−1^), mixed region (from 1,500 to 1,200 cm^−1^), polysaccharide region (from 1,200 to 900 cm^−1^), true fingerprint region (from 900 to 700 cm^−1^), and the full range of the spectra (from 3,000 to 700 cm^−1^).

The training data set was used to construct a predictive model based on the random forest (RF) algorithm, which was trained to classify the isolates based on their STs. The training process used a *k*-fold method (*k* = 10) for hyperparameter tuning, including the number of estimators, maximum features, maximum depth, minimum split size, and minimum samples per leaf. The second derivative was used for analysis. Accuracy and balanced accuracy scores were determined for all isolates included in the validation assay and for each ST category. Both resulting prediction models (one for each center) were subsequently subjected to cross validation. Area under receiver operating characteristic curves (AUROC) were reported as performance metrics. The AUROC can be understood as the probability of correctly classifying an infrared spectrum into the correct category (25). An AUROC value > 0.9 is considered to indicate excellent performance, values between 0.7 and 0.9 indicate good performance, and values less than 0.7 indicate suboptimal performance.

#### Intra-center repeatability and inter-center reproducibility assays

The same repeatability assay was conducted in both centers to assess the robustness of intra-center acquisition. The average spectrum for each isolate was compared with the respective triplicate spectra, resulting in calculation of the differentiation index (*D*) for each sample in both centers, as *D* = (1 − r)∙1,000 (where *r* is Pearson’s coefficient), for the regions of the spectra and combinations of regions described in the previous section. The differentiation index, *D*, ranges from 0 to 2,000, where 0 indicates identical spectra, 1,000 indicates noncorrelated spectra, and 2,000 indicates negatively correlated spectra (26). The value of *D* was calculated as the mean and median using the second derivative of the spectra.

An inter-center reproducibility assay was also performed to evaluate the variability between centers arising from external factors such as atmospheric conditions, configuration of the instruments, calibration, and the person involved in the spectra acquisition. For this purpose, average spectra from each center were compared pairwise with the corresponding average spectra from the other center. This comparison enabled calculation of the differentiation index (*D*) for each pair of samples acquired between the two centers. The value was calculated as the mean and median using the second derivative of the spectra.

## RESULTS

### Genome-based epidemiological analysis

The prevalence rates of carbapenemase genes among the *K. pneumoniae* isolates were as follows, OXA-48-like (*n* = 311, 85.21%), KPC-3 (*n* = 39, 10.68%), NDM-like (*n* = 7, 1.92%), VIM-1 (*n* = 6, 1.64%), and two double carbapenemases (NDM-1 and KPC-3 plus OXA-48) (Table S1).

**Fig 2:**
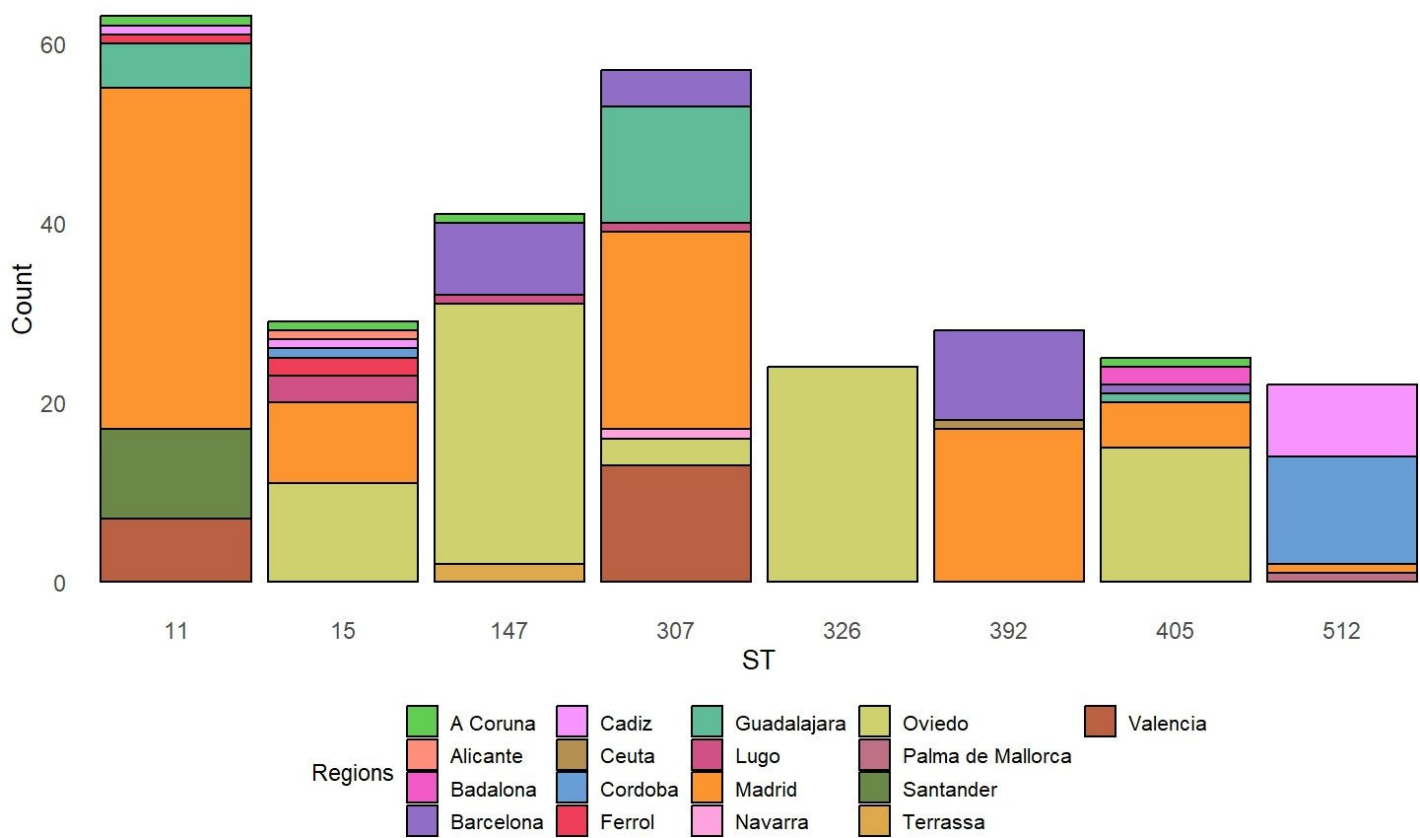
Distribution of the carbapenemase-producing *K. pneumoniae* isolates by ST and geographical distribution in regions.

Regarding the population structure of carbapenemase-producing *K. pneumoniae*, the following clones were included in this study: ST11 (*n* = 63, 21.80%), ST307 (*n* = 57, 19.72%), ST147 (*n* = 41, 14.19%), ST15 (*n* = 29, 10.03%), ST392 (*n* = 28, 9.69%), ST405 (*n* = 25, 8.65%), ST326 (*n* = 24, 8.30%), and ST258/512 (*n* = 22, 7.61%) (Fig. 2). The most widespread clone, ST11, was distributed in the north and center of Spain, while the other major clone, ST307, was mainly located in the center of the country. The presence of ST326 exclusively in Oviedo, in the north of Spain, was of note (27). Globally, ST11, ST14, ST101, ST147, and ST258/512 are the major carbapenemase-producing *K. pneumoniae* clones (28). The findings of the present study are partly consistent with the previous data, highlighting the presence of ST11, ST147, ST307, and ST512. In the present study, we observed a large spread of ST11 and ST15 between regions, along with ST307, ST405, ST147, and ST512, which were isolated in three or more different regions. The inter-regional spread of these five clones in Spain indicates that they are at epidemiological stage 4 (multiple epidemiologically related outbreaks occurring in different health districts, indicating inter-regional autochthonous inter-institutional transmission).

The analysis of 289 contigs across various STs revealed a diverse distribution of plasmid replicase types associated with carbapenemase genes (Table S2). Notably, IncL/M was the most prevalent replicase type, present across multiple STs (11, 15, 147, 307, 326, 392, and 405). Other common types included IncFIB and IncR. Of the analyzed contigs, 68.86% (199/289) was associated with plasmids, suggesting a significant role of plasmids in carbapenemase dissemination. However, these results must be interpreted cautiously due to two main limitations: potential chromosomal location and incomplete genome assemblies.

SNP-based comparison of all sequenced *K. pneumoniae* genomes was conducted and enabled assignment of the strains into 31 WGS clusters (designated from “A” to “EE”). Clusters were grouped together when <18 SNPs of difference are found among isolates. These groups each included a different number of isolates, ranging from 2 to 13. The varying degree of how these isolates were related to one another can be observed in the phylogenetic tree (Fig. S1). Considering these findings together, the distribution of the isolates reflects the great diversity of *K. pneumoniae* strains. This indicates that the data were not obtained as part of an outbreak surveillance but actually comprise the actual distribution of carbapenemase-producing *K. pneumoniae* in Spain. Thus, roughly half of the 289 *K*. *pneumoniae* isolates (153/289, 53%) were classified as singletons by WGS clonal analysis.

**Fig 3:**
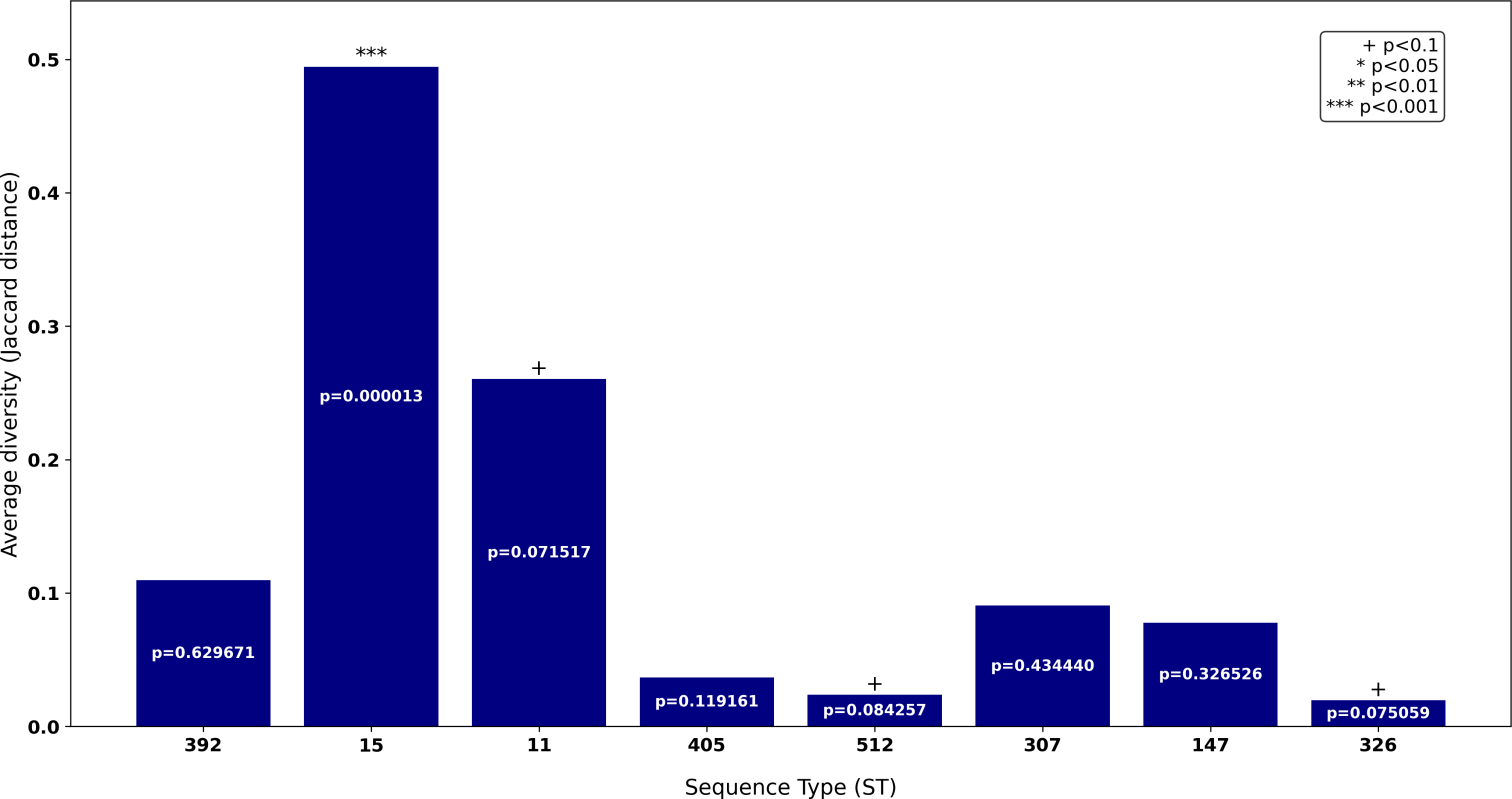
Intra-ST diversity of polysaccharide-related accessory genes in *K. pneumoniae*. Bars represent the average Jaccard distance between genetic profiles within each ST. Statistical significance was determined using a t-test: ^+^*P* < .1 (marginally significant), *P < 0.05 (significant), **P < 0.01 (highly significant), and ***P < 0.001 (extremely significant).

The pangenome analysis of *Klebsiella pneumoniae* identified 7,391 genes in the accessory genome. Of these, 127 genes (1.72%) were associated with the encoding of polysaccharide-related proteins, based on the presence of 14 specific domains identified in the Pfam database. The presence of polysaccharide-related genes differs among the different ST, and thus, to investigate whether this variability could be related to the accuracy of the FTIR classification, we analyzed the intra-ST diversity of the polysaccharide-related genes. A histogram was generated to visualize the distribution of average intra-ST Jaccard distances for each ST. The histogram, Fig. 3, was created using the Matplotlib library in Python (version 3.8). ST15 exhibited the highest intra-ST diversity, with an average Jaccard distance of 0.49 and a very high statistical significance (*P* < 0.001). This suggests a high variability in the polysaccharide gene profiles within this ST. ST11 displayed the second highest intra-ST diversity (0.26), although it is reduced by half with respect to ST15. In contrast, ST307 has an average Jaccard distance of 0.11, and in the opposite limit, ST326 showed the lowest intra-ST diversity, with an average Jaccard distance of approximately 0.02 and marginal significance (*P* < 0.1), indicating greater homogeneity in the polysaccharide gene profiles.

### FTIR spectroscopy-based typing

For FTIR clustering of all carbapenemase-producing *K. pneumoniae*, an optimal cut-off value of 0.497 was established after including the information regarding the ST of the isolate as metadata (Fig. S2). The cophenetic correlation coefficient was 0.865, indicating that the isolates belonging to the same cluster are very similar. Among all 289 carbapenemase-producing *K. pneumoniae* tested, FTIR detected 12 clusters (namely, I–XII, including 288 isolates) and 1 singleton. The two largest clusters were cluster I (*n* = 62, 21.5%) and cluster IV (*n* = 51, 17.6%).

The concordance between FTIR and the ST classification obtained by WGS was as follows: FTIR grouped in the same cluster isolates of different STs on seven occasions (clusters I, III, IV, V, VI, VIII, and XII). Nevertheless, perfect agreement between ST and FTIR was observed in five cases (clusters II, VII, IX, X, and XI). The AWI enabled us to explore the potential limit of FTIR as an epidemiological control tool in a suspected outbreak scenario or in everyday routine work and to examine how genome composition may infer FTIR classification. This yielded an AWI value of **0.611** (95% CI, 0.531–0.680) for FTIR clustering considering MLST *in silico* as the reference method. Thus, approximately 61% of the isolates involving an ST cluster was grouped in the same way as in FTIR clustering. These findings show a moderate level of similarity between the methodologies.

The concordance scores can be better understood by examining the contingency table (Table 1). Notably, the accuracy levels for ST prediction were highest for ST307 and ST326 (89% and 87%, respectively). The opposite was observed for ST15 and ST11, for which the classification accuracy was lowest (24% and 59%, respectively). Moreover, none of the STs were exclusively grouped in a single FTIR cluster in any case, as it is the case for the aforementioned ST11, which was identified in the largest number of isolates (*n* = 63) and was subgrouped in four FTIR clusters (I, IV, V, and VI), and in one case, it was also deemed as the only singleton identified by FTIR.

**TABLE 1:** Contingency table showing relationship between ST and FTIR clustering

ST	FTIR													Total	Accuracy^*b*^ (%)
	I	II	III	IV	V	VI	VII	VIII	IX	X	XI	XII	S^*a*^		
512						4				18				22	81
326		21				2						1		24	87
405	1		1		1	2						20		25	80
392	1		1			4			22					28	78
15	7			12		3	5				2			29	24
147			1	1		4		35						41	74
307	51			1		4		1						57	89
11	2			37	7	16							1	63	59
Total	62	21	3	51	8	39	5	36	22	18	2	21	1	289	
Accuracy**^*c*^** (**%**)	82	100	33	72	88	41	100	97	100	100	100	95	100		

^*a*^ Singleton.

^*b*^ The accuracy was measured by considering the ST of the isolate as the reference measure; the result thus indicates how well the ST is correctly predicted within a unique major FTIR cluster.

^*c*^ The accuracy was measured by considering FTIR clustering as the reference method; the result thus indicates how well the FTIR cluster predicts that an isolate will belong to a unique major ST group.

For FTIR clustering of all carbapenemase-producing *K. pneumoniae*, an optimal cut-off value of 0.411 was established after including the information regarding the clonal cluster obtained by the WGS analysis of the isolate as metadata (Fig. S3). Among all 289 carbapenemase-producing *K. pneumoniae* tested, FTIR detected 14 clusters (namely, I–XIV, including 287 isolates) and 2 singletons. The largest cluster was cluster I (*n* = 26, 19.1%).

To further evaluate the benefit of enhancing epidemiological genome-based analysis by FTIR, we examined which of the isolates that formed part of a clonal cluster (≤18 cgMLST) were grouped by FTIR, yielding an AWI value of 0.244 (95% CI, 0.185–0.304), whereas the AWI value comparing the clustering by FTIR versus WGS clonal clustering was 0.652 (0.474–0.612 IC95). The disparity in AWI concordance indicates that numerous small WGS groups were included within a single FTIR clonal cluster. This is evident in the contingency table of clonal relatedness (Table 2), wherein small WGS clonal clusters (from A to R) were consistently located within a single FTIR cluster. However, these FTIR clusters often also included other WGS clonal clusters, as demonstrated by their recurrence within FTIR clusters I, VI, XII, and XIV. To establish the AWI concordance, singletons were excluded from the clustering comparison. Some of the major WGS clonal groups (denoted from AA to EE) showed varying degrees of classification in a unique FTIR cluster. The only FTIR clonal groups, which were correlated 100% with the WGS clonal groups, were FTIR clusters III, IV, and X. Accuracy values are not provided for all groups due to the small number of isolates in each cluster.

**TABLE 2:** Contingency table between FTIR clustering and WGS clustering based on clonal and phylogenetic relatedness (≤18 SNPs within a unique monophyletic group)

WGS clonality clustering	FTIR																Total^*a*^
	I	II	III	IV	V	VI	VII	VIII	IX	X	XI	XII	XIII	XIV	S1^*c*^	S2	
A	2																2
B												2					2
C												2					2
D												2					2
E														2			2
F														2			2
G													1			1	2
H											2						2
I											2						2
J									2								2
K												2					2
L						1			1								2
M						2											2
N						2											2
O							3										3
P								3									3
Q	3																3
R									3								3
S			2							2							4
T									2		3						5
U				5													5
V	5																5
W	5																5
X				6													6
Y					1				1					6			8
Z													8				8
AA						9											9
BB									1		8						9
CC									2			6			1		9
DD		1				6			3								10
EE	11								2								13
Total^*b*^^*b*^	26	1	2	11	1	20	3	7	17	2	15	14	9	10	1	1	136

^*a*^ Total number of isolates correctly predicted within a unique major FTIR cluster when applying the clonal cluster (<18 SNPs by cgMLST) of the isolate as the reference measure.

^*b*^ Total number of isolates correctly predicted within a unique major clonal cluster (<18 SNPs by cgMLST), when applying the FTIR clustering as the reference method.

^*c*^ Singleton.

### Improvement of FTIR analysis by use of AI tools

We attempted to improve the discrimination and clustering of FTIR in the data set by using AI tools. We used the IR Biotyper software and three supervised models (ANN, SVM linear, and SVM RBF) to determine whether or not a spectrum belonged to the corresponding ST. Comparison with the clonal clusters in the same phylogenetic group was not possible using supervised models because the small number of isolates included the same clonal cluster owing to the high variability in the target bacterial population. The training data set (*n* = 197) was randomly split into 4 parts (*k* = 4), each of which contained 25% of the spectra and was used as a test set after training the ANN with the remaining 75% of the spectra generated. One experiment consisted of four iterations of training/testing, so that each pair of spectra was included in the test set without having been encountered by the ANN during training. Four experiments were conducted to determine the average performance of the ANN, and the same procedure was also conducted with the other algorithms: SVM linear and SVM RBF. The accuracy of the FTIR analysis performed in the HGM center at the training stage ranged from 81.0% to 84.0%, while in the validation stage, it ranged from 71.7% to 81.5% depending on the method and the validation center considered (Table 3). The best results were obtained with the ANN algorithm, with an average accuracy of 80.5%. The results of the training and validation performed in parallel in the HUCA center are outlined in Table S3. The most accurate prediction was obtained for ST307 (94%). The opposite was observed for ST15, ST392, and ST405, for which the classification accuracy was lowest, with values of 55.6%, 55.5%, and 54.2%, respectively, for validation and 66.6%, 55.5%, and 58.3%, respectively, for cross validation.

**TABLE 3:** Typing results of FTIR spectroscopy by using AI in the IR Biotyper software

Algorithm^*a*^	Global accuracy %				Accuracy by ST %															
	Training HGM (*n* = 197)	Validation (*n* = 92)			ST11 (23)		ST15 (6)		ST147 (13)		ST307 (18)		ST326 (8)		ST392 (9)		ST405 (8)		ST512 (7)	
		HGM^*b*^	HUCA^*c*^	Average	HGM	HUCA	HGM	HUCA	HGM	HUCA	HGM	HUCA	HGM	HUCA	HGM	HUCA	HGM	HUCA	HGM	HUCA
ANN	81.0	79.4	81.5	80.5	91.3	91.3	33.3	33.3	84.6	92.3	94.4	94.4	75.0	75.0	55.5	55.5	62.5	75.0	85.7	85.7
SVM linear	84.0	71.7	71.7	71.7	60.9	56.5	66.7	83.3	84.6	84.6	94.4	94.4	75.0	75.0	55.5	55.5	50.0	50.0	71.4	71.4
SVM RBF	83.0	76.1	73.9	75.0	73.9	65.2	66.7	83.3	84.6	84.6	94.4	94.4	75.0	75.0	55.5	55.5	50.0	50.0	85.7	71.4
Overall	82.7	75.7	75.7	75.7	75.4	71.0	55.6	66.6	84.6	87.2	94.4	94.4	75.0	75.0	55.5	55.5	54.2	58.3	80.9	76.2

^*a*^ For FTIR clustering in the IR Biotyper software, the following AI methods were used: ANN, SVM linear, and SVM RBF, with a *k*-fold cross validation method (*k* = 4).

^*b*^ HGM, Hospital General Universitario Gregorio Marañón.

^*c*^ HUCA, Hospital Universitario Central de Asturias.

Regarding the scores obtained in the validation stage using the ANN classification algorithm, 63 isolates (68%) yielded a score below 1.0, indicating high reliability, 19 isolates (21%) yielded a score between 1 and 2, and 10 isolates (11%) yielded a score above 2, indicating the low reliability of the result. Among the results of low reliability, 8 of the 10 isolates belonged to ST11, 6 were correctly assigned to ST11, 1 was assigned to ST15, and the last 1 to ST326. The other 2 isolates with results of low reliability belonged to ST307 and ST407, both wrongly assigned to ST11 by FTIR and thus implying that the classification of ST11 was not reliable by FTIR. Complete data are provided in Table S3.

Regarding the Clover MSDAS software, we used the RF algorithm to determine whether or not a spectrum belonged to the corresponding ST categorized by sequence typing. Data regarding the membrane lipid region (from 3,000 to 2,800 cm^−1^), the protein region (from 1,800 to 1,500 cm^−1^), and the mixed region (from 1,500 to 1,200 cm^−1^) yielded worse results in the training and were therefore not included in the validation analysis. Overall, the best results were obtained for the polysaccharide region (Table 4), closely followed by the full range of the spectra. However, this latter region yielded worst results in the cross validation studies between centers, and therefore, the polysaccharide region was chosen as reference for further studies. Complete data can be consulted in Table S4.

**TABLE 4:** Typing results of FTIR by using the RF prediction model in the Clover MSDAS software using different wavelength regions and the second derivative

Wavelength region used in FTIR^*a*^	Accuracy %^*b*^ (*k*-10-fold)		
	Training HGM^*c*^ (*n* = 197)	Validation HGM (*n* = 92)	Validation HUCA^*d*^ (*n* = 92)
Lipids	62.39	–^*e*^	–
Proteins	52.30	–	–
Mix	81.35	–	–
Polysaccharides	86.78	75.25	75.67
True fingerprint	82.86	73.14	75.35
Full range	82.14	74.32	76.28

^*a*^ For FTIR clustering in the Clover MSDAS software, the RF model was applied using the following optimized hyperparameters; estimators 100, maximum features 17, maximum depth 10, minimum split size 2, and minimum samples per leaf 1. The absorbance bands of the spectra were filtered as the membrane lipid region (from 3,000 to 2,800 cm^−1^), protein region (from 1,800 to 1,500 cm^−1^), mixed region (from 1,500 to 1,200 cm^−1^), polysaccharide region (from 1,200 to 900 cm^−1^), true fingerprint region (from 900 to 700 cm^−1^), and the full range of the spectra (from 3,000 to 700 cm^−1^).

^*b*^ Accuracy levels are expressed in %.

^*c*^ HGM, Hospital General Universitario Gregorio Marañón.

^*d*^ HUCA, Hospital Universitario Central de Asturias.

^*e*^ –, no further validation studies have been performed for these regions of the spectrum.

Balanced accuracy is the recommended parameter as it takes into account the number of isolates into each category, which is particularly useful when dealing with imbalanced data, as in this case. The balanced accuracy in the training stage was 86.78% while in the validation stage, it was 75.25% for validation and 75.67% for cross validation (Table 5). The accuracy was almost the same for the validation and cross validation analyses, except for ST11 and ST147, highlighting the robustness of the method regardless of the center, equipment, operator, and other experimental conditions.

**TABLE 5:** Typing results of FTIR by using the RF prediction model in the Clover MSDAS software using the polysaccharide region and the second derivative

ST/FTIR clustering^*a*^ (*n*/*n*)^*b*^	Accuracy %^*c*^ (k-10 fold)			AUROC^*d*^		
	Training HGM^*e*^ (*n* = 197)	Validation HGM (*n* = 92)	Validation HUCA^*f*^ (*n* = 92)	Training HGM	Validation HGM	Validation HUCA
ST307 (39/18)	89.74	94.44	94.44	0.95	0.98	0.97
ST326 (16/8)	100.00	75.00	75.00	0.99	0.96	0.94
ST392 (19/9)	94.74	55.56	55.56	0.97	0.94	0.82
ST405 (17/8)	94.12	62.50	62.50	0.95	0.93	0.96
ST512/258 (15/7)	86.67	85.71	85.71	0.96	0.98	0.99
ST11 (40/23)	87.50	60.87	56.52	0.97	0.93	0.92
ST147 (28/13)	89.29	84.62	92.31	0.99	0.94	0.97
ST15 (23/6)	52.17	83.33	83.33	0.84	0.87	0.93
Total	86.78^*g*^	75.25	75.67	0.95	0.94	0.94

^*a*^ For FTIR clustering in the Clover MSDAS software, the RF model was applied using the following optimized hyperparameters; estimators 100, maximum features 17, maximum depth 10, minimum split size 2, and minimum samples per leaf 1.

^*b*^ Number of isolates in the training/validation set.

^*c*^ Accuracy levels are expressed in %.

^*d*^ AUROC, area under receiver operating characteristic curves.

^*e*^ HGM, Hospital General Universitario Gregorio Marañón.

^*f*^ HUCA, Hospital Universitario Central de Asturias.

^*g*^ The total is expressed as balanced accuracy.

Slightly higher values were obtained with the ANN analysis in the IR Biotyper software than with the RF algorithm in the Clover MSDAS software, using the polysaccharide region. Comparison of these results with those obtained by unsupervised methods showed that the classification accuracy increased from 61% of the PCA to 75.0% for the RF algorithm in the Clover MSDAS software and up to 81% for the ANN algorithm in the IR Biotyper software. Hence, AI algorithms are good options for analysis of infrared spectra for typing purposes. Notably, the highest accuracy of ST prediction was obtained for ST307, with 94.44% in both centers, overlapping with the PCA results. The opposite was observed for ST11 and ST392, for which the classification accuracy was lowest for validation, 60.87% and 56.52%, respectively, and for cross validation, 56.56% and 55.56%, respectively. The low accuracy was obtained with both unsupervised and supervised classification models. However, the accuracy of the clustering of ST15, which was poorly identified by PCA (24% accuracy), increased notably (up to 83.33%) in both validation and cross validation analyses. Notably, this was not observed in the training stage. These discrepancies in the classification of some STs may be due to the lower number of isolates in the validation stage relative to the unsupervised methods or to the higher capacity of the algorithms to recognize certain characteristics in the infrared spectra. On comparing both types of analytical software and AI methods, we observed a higher prediction accuracy for ST15 with the Clover MSDAS software using the RF algorithm and the opposite for ST11, prediction of which was very much improved with the IR Biotyper software using the ANN method. This difference cannot be directly attributed to the performance of the classifying method; as in the case of the ST15 and the use of the RF algorithm in the Clover MSDAS software, the training accuracy was low, while the validation accuracy was high, probably due to the smaller number of isolates in the validation set than in the training set. In the case of ST11 and use of the IR Biotyper software with the ANN method, we observed excellent accuracy of prediction, particularly in comparison with the other methods. However, the scores revealed poor-quality assignment of the spectra, and the data must therefore be interpreted with caution.

The AUROC indicated that the training stage proved excellent for classifying all ST except ST15 (Table 5). This was also observed at the validation stage, with a global AUROC of 0.95, demonstrating a high overall performance of the method. The RF algorithm applied to FTIR spectra is therefore capable of providing precise epidemiological information. The classification was poorer in the validation stage for ST392, being the AUROC value considered good. For cross validation, classification results were even better for ST15, increasing AUROC values from 0.87 to 0.93. No AUROC values below 0.8 were obtained in any case. The AUROC are represented in Fig. S4.

### Repeatability and reproducibility of the workflow

**Fig 4:**
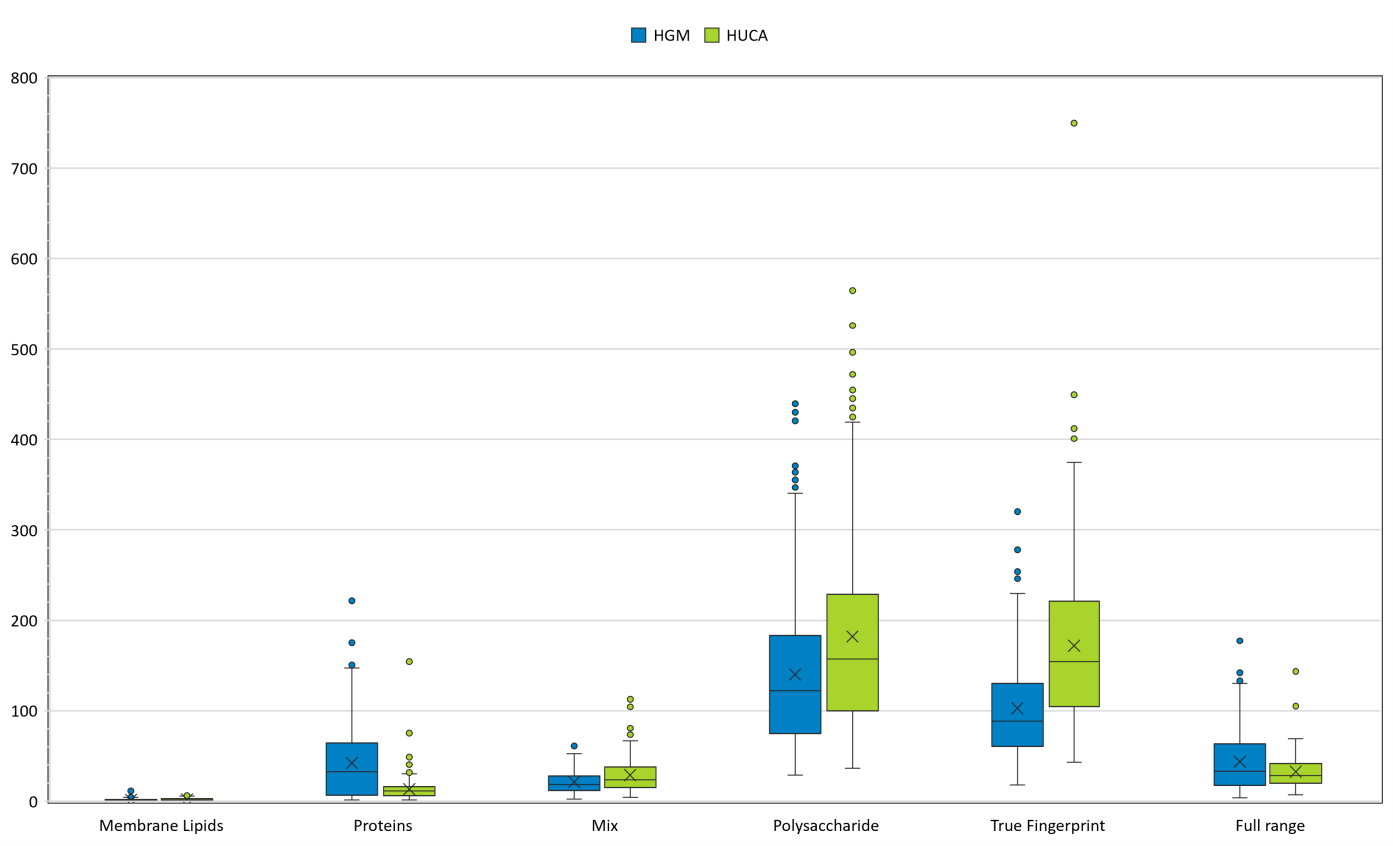
Repeatability results expressed by the *D* value in both HGM and HUCA centers, for the different infrared spectral ranges.

Repeatability was assessed by testing the average spectra from each isolate relative to the corresponding replicates, so that the same experimental conditions (operator, culture, and FT-IR spectra acquisition) were experienced. The lowest *D* values corresponded to the lipid region, while the highest *D* values corresponded to the polysaccharide region, indicating that the lipids were stably expressed in the different samples, while the polysaccharides were more variable (Fig. 4). This was also observed in data scattering, which was higher for the polysaccharides and for the true fingerprint region. The complete data set can be provided by the authors upon request.

**Fig 5:**
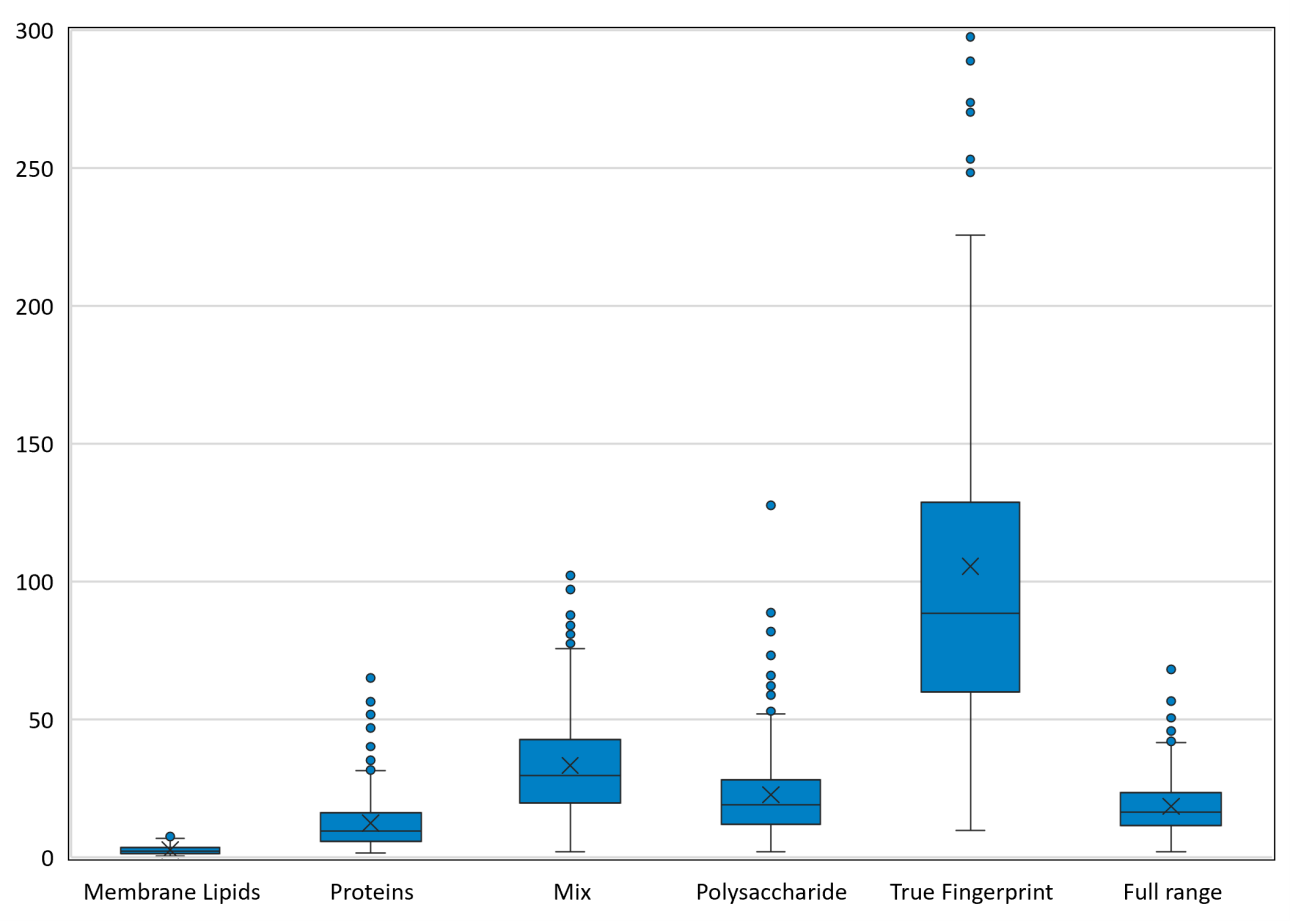
Reproducibility results expressed by the *D* value for the different infrared spectral ranges when comparing the HGM and HUCA centers.

Reproducibility was assessed by testing the average spectra obtained by one center in pairwise comparison with the corresponding average spectra from the other center, to evaluate the influence of the experimental conditions. The lowest *D* values corresponded to the lipid region, indicating that the lipids were stably expressed in both centers, while the true fingerprint region provided the highest *D* values, reflecting a high degree of variability and therefore being excluded as an optimal region for clustering in a global networking environment (Fig. 5). The D value was very similar for the protein, mixed, and polysaccharide regions. Data scatter was minimal for the lipid region of the spectra and for the repeatability analysis. The Pearson correlation coefficient is 0.98 reflecting very highly correlated measurements between both centers. The complete data set is provided in Table S5.

## DISCUSSION

In this study, we evaluated the performance of FTIR spectroscopy as a rapid typing tool for the surveillance of carbapenemase-producing *K. pneumoniae*. Although other studies have used this technique for typing several bacterial species, they have generally relied on clinical isolates collected during an outbreak. By contrast, we examined a collection of isolates that are highly relevant in terms of infection control measures but obtained with the aim of mimicking an endemic situation where all types of possible transmission events may occur.

We found that FTIR spectroscopy together with unsupervised algorithms can measure the diversity of carbapenemase-producing *K. pneumoniae* under surveillances. The AWI value obtained from measuring the agreement between FTIR and WGS (considered the reference method) was 0.611 when the classification was made according to the ST and 0.652 for the clonal clustering obtained by cgMLST. Although these values are slightly lower than reported in previous studies (7, 8), other methodological strengths may have led to the lower concordance. First, we analyzed isolates collected from more than one hospital location, from different patients, and over an extended period of time (from 2014 to 2019), mimicking the present endemic situation of carbapenemase-producing *K. pneumoniae* in the clinical settings of the south of Europe and other areas (29), while previous studies have analyzed isolates from a single hospital location, collected during an unique event or outbreak. Second, our study included more isolates than in previous studies of carbapenemase-producing Enterobacterales, encompassing a great diversity even within the same ST. This was particularly evident for ST11 and ST15, the most widely distributed of the STs under study. ST11, which is the most numerous ST (*n* = 63), also has the most epidemiologically unrelated events represented by seven different WGS clonal clusters (L, M, N, P, R, AA, and DD). The lowest levels of accuracy values were therefore obtained for ST11 (24%) followed by ST15 (59%). The genome composition of *K. pneumoniae* in larger clones may be challenging to identify, with large differences between isolates. The opposite was observed for ST307 and ST326, which are not as widely geographically distributed, so that higher AWI values (89% and 87%, respectively) were obtained. While ST307 (*n* = 57) is composed of five clonal groups, it evidently performs better for FTIR classification. This suggests that ST11 may encompass distinguishable phenotypes detectable by FTIR, leading to a lower classification accuracy than for ST307. Thus, FTIR should perform better for isolates that are phylogenetically closer. We also conducted cgMLST phylogenetic analysis in order to improve the resolution of the larger clusters (ST11 and ST15) and to check whether FTIR could yield the same level of accuracy in the classification. The concordance revealed an AWI of 0.652, being close to the previous value within the classification by ST, thus indicating excellent performance. In this case, similar and small WGS clonal clusters were included in a single FTIR cluster, highlighting the level of differentiation that can be achieved.

To further elucidate the reason why some STs behave better than others in the FTIR classification, we conducted an in-depth analysis of the polysaccharide-associated genes in the accessory genome, as it is the default region of the spectra used by FTIR for clustering. We observed an inverse relationship between the intra-ST diversity of the polysaccharide-related accessory genes and the accuracy of FTIR classification. ST15, which showed the highest intra-ST diversity, presented one of the lowest accuracy values in FTIR clustering, especially in nonsupervised methods of analysis. The high variability in the polysaccharide gene profiles within this ST suggests genetic heterogeneity that could result in a wide range of polysaccharide phenotypes, hindering accurate classification by FTIR, and could explain its tendency to be confused with other STs in FTIR classification. On the contrary, the low variability and greater homogeneity in polysaccharide gene profiles could be translated into more consistent phenotypes and more accurate FTIR classification, as in ST326 or ST512. In conclusion, intra-ST diversity appears to influence the phenotypic expression of polysaccharides, affecting the ability of FTIR to distinguish between different STs.

Moreover, and to support our conclusions, 309 out of the 365 isolates included in the study are OXA-48 producers; thus, the presence of this carbapenemase is predominant in our population. A side effect of this predominance is that the clustering of FTIR should not be influenced by the resistance mechanisms themselves, but rather exclusively by the epidemiological links among the isolates.

Regarding the cut-off value in the dendogram, in our study, we used a value of 0.497 for ST comparisons and 0.411 for clonal cluster (obtained by cgMLST) comparisons for the isolate. These values were higher than those observed in previous studies. However, notable discrepancies related to the cut-off values also emerged in previously published studies (7–9), which can also be attributed to the strong influences of the microorganism, sample size, the purpose of the analysis (i.e., typing an isolate during an outbreak or during surveillance), and even the standard of comparison. Besides, we can justify the quality of the data by the scores (68% expressing high reliability) obtained with the IR Biotyper software. For all these reasons, in our opinion, establishing a standard cut-off value is not appropriate practice and cut-offs should only be established after internal validation and considering the target population and the study aims, as also recommended by the manufacturer.

Moreover, we have demonstrated that supervised methods can improve the recognition of carbapenemase-producing *K. pneumoniae* isolates, yielding values ranging from 80.5% of the ANN in the IR Biotyper software method to 75.0% of the RF method in the Clover MSDAS software. The values also varied depending on the ST, with the clustering of ST11 again being poor. ST307 was the ST most accurately identified by all algorithms evaluated. Likewise, those ST yielding accuracy values lower than 80% all belong to three or more clusters, that is, ST393, ST405, and ST11. Besides, all those are geographically widespread, revealing higher levels of intrinsic variability. Curiously, ST307 behaves better in classification when compared with ST393, while both include five cgMLST clusters and ST307 is distributed more widely in the geographical area studied. Furthermore, ST307 *K. pneumoniae* traditionally exhibits hypermucous phenotypes that could lead to misidentifications if evolutionary events involving the capsule take place *in vivo*. However, our results prove no inconsistencies among this serotype (22, 23). To note, ST392 is mainly found in Madrid and Barcelona; these cities are the biggest in Spain and bring together the largest number of reference hospitals, representing scatter population units.

The AUROC values for the RF algorithm (above 0.9 indicating excellent classification) highlight the outstanding predictive performance of AI classifiers. No inconsistencies were found between the IR Biotyper and the Clover MSDAS software with respect to the classification by ST, except for ST15, which was better identified by the Clover MSDAS software. However, due to the low number of isolates in the validation stage (*n* = 5), this conclusion may be taken with caution. In conclusion, we observed that, as in unsupervised methods, more complex data, subject to variations in sample size, geographical origin, and even biological differences (due to the ongoing evolution of the local microbial populations), will affect the prediction task.

We also found some minor inconsistencies between unsupervised and supervised models, e.g., the clustering of ST392 was worse with supervised than with the unsupervised classifiers. Notably, all samples are used for analysis in unsupervised classification methods, while in supervised methods, the isolates are split intro training and validation stages, which may affect the classification, as the algorithm is limited by the smaller amount of information. A prerequisite of supervised models to work at the highest level is to construct large-spectra databases for training. It is therefore likely that further inclusion of more isolates in the database will provide better results. Larger-scale validation studies are therefore needed to optimize spectral databases and/or models and to continue improving typing results with FTIR spectroscopy.

In contrast to usual practice, we upgraded the manufacturer’s default regions of analysis in the spectra, including not only the polysaccharide region but other regions, such as the lipid and the protein regions, and studied the possible influence of each in the final clustering. We support the use of the polysaccharide region for FTIR typing as this yielded the best performance in terms of classification using supervised models, reflected by the compromise between repeatability and reproducibility for the different centers. We hypothesize that although the lipid region yielded lower *D* values for repeatability and reproducibility, this area may be so uniform among bacteria that prevent accurate differentiation and clustering among epidemiological unrelated isolates.

We also demonstrate that translation of FTIR spectroscopy into routine work in microbiology laboratories is possible. Several authors have reported difficulties in standardization with a lack of reproducibility between equipment (30, 31). We performed cross validation studies between two centers (HGM and HUCA), with two different types of equipment and different operators providing cross validation AUROC with an average value higher than 0.9, thus providing an excellent ability of prediction and endorsing the possibility of constructing universal spectral libraries that could be easily shared among users and facilitating the expansion of current databases for other lineages. Updates regarding the institution or the geographic origin could easily be achieved. Besides, the use of flexible, user-friendly, and integrated platforms, as the IR Biotyper software, will facilitate adoption in the clinical setting and provide opportunities to consolidate real-time applications at a global level. However, prospective clinical studies involving multiple sites with a different prevalence of antimicrobial-resistant bacteria and different high-risk clones should be conducted for full evaluation of the clinical impact.

In conclusion, FTIR spectroscopy is a rapid, inexpensive typing method that can easily be integrated in routine clinical microbiology laboratories and can support the identification of high-risk clones in carbapenemase-producing *K. pneumoniae* and other Enterobacterales. Use of the method will facilitate general surveillance of these clones as well as their identification in outbreak situations, by reducing the workload and time traditionally spent in typing, preserving WGS for confirmation and further characterization.

## SUPPLEMENTAL MATERIAL

Figure S1 jcm.01122-24-s0001.pdf Phylogenetic tree obtained from cgSNP analysis of carbapenemase-producing *K. pneumoniae* isolates showing identified clusters.

Figure S2 jcm.01122-24-s0002.png FTIR clustering of all carbapenemase-producing *K. pneumoniae* related to the ST of the isolate included as metadata.

Figure S3 jcm.01122-24-s0003.png FTIR clustering of all carbapenemase-producing *K. pneumoniae* related to the clonal cluster of the isolate included as metadata.

Figure S4 jcm.01122-24-s0004.pdf ROC curves.

Supplemental legends jcm.01122-24-s0005.docx Legends for Fig. S1 to S4.

Table S1 jcm.01122-24-s0006.xlsx Description of the 365 unduplicated carbapenemase-producing *K. pneumoniae* clinical isolates.

Table S2 jcm.01122-24-s0007.xlsx Distribution of plasmid replicase types across different sequence types (STs).

Table S3 jcm.01122-24-s0008.xlsx Results of the supervised methods in the IR Biotyper software.

Table S4 jcm.01122-24-s0009.xlsx Results of the validation obtained in the Hospital General Gregorio Marañón (HGM) and in the Hospital Universitario Central de Asturias (HUCA) when using the Random Forest (RF) algorithm in the Clover MSDAS software and the training FTIR database obtained in the HGM.

Table S5 jcm.01122-24-s0010.xlsx D values corresponding to the FTIR spectrum of each isolate in one centre in pairwise comparison with the same isolate in the other centre.

ASM does not own the copyrights to Supplemental Material that may be linked to, or accessed through, an article. The authors have granted ASM a non-exclusive, world-wide license to publish the Supplemental Material files. Please contact the corresponding author directly for reuse.
