# Supplementary figures and images for: Multicenter evaluation of Fourier transform infrared (FTIR) spectroscopy as a first-line typing tool for carbapenemase-producing Klebsiella pneumoniae in clinical settings
Source: J Clin Microbiol. 2024 Nov 27;63(1):e01122-24. doi: 10.1128/jcm.01122-24 (PMC11784409; doi:10.1128/jcm.01122-24)

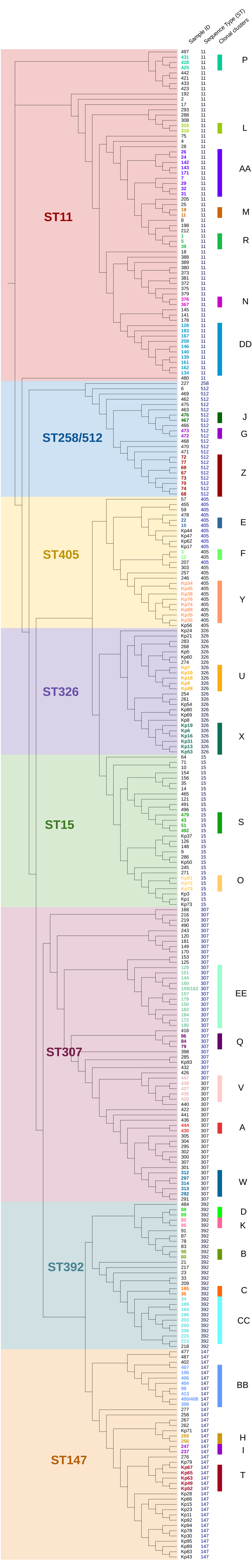

Supplement: Figure S1 — Phylogenetic tree obtained from cgSNP analysis of carbapenemase-producing K. pneumoniae isolates showing identified clusters. [file jcm.01122-24-s0001.pdf]

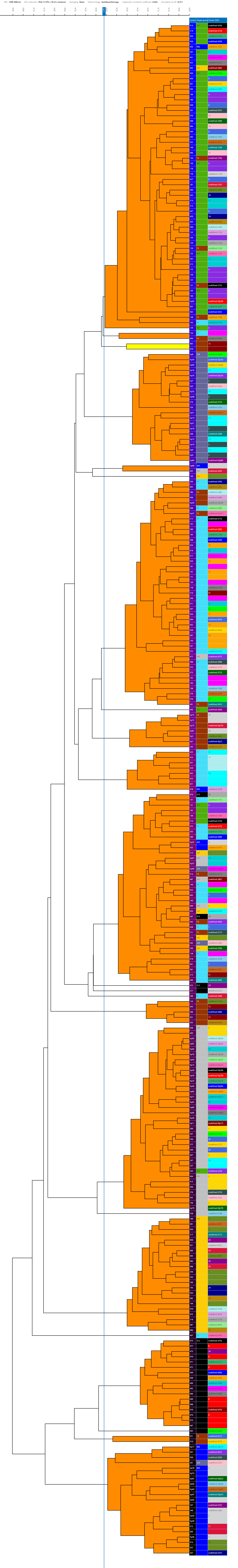

Supplement: Figure S3 — FTIR clustering of all carbapenemase-producing K. pneumoniae related to the clonal cluster of the isolate included as metadata. [file jcm.01122-24-s0003.png]
