# Supplementary material for: Multicenter evaluation of Fourier transform infrared (FTIR) spectroscopy as a first-line typing tool for carbapenemase-producing Klebsiella pneumoniae in clinical settings
Source: J Clin Microbiol. 2024 Nov 27;63(1):e01122-24. doi: 10.1128/jcm.01122-24 (PMC11784409; doi:10.1128/jcm.01122-24)

A. AUROC representation when the training was performed in HGM and the validation in the HGM

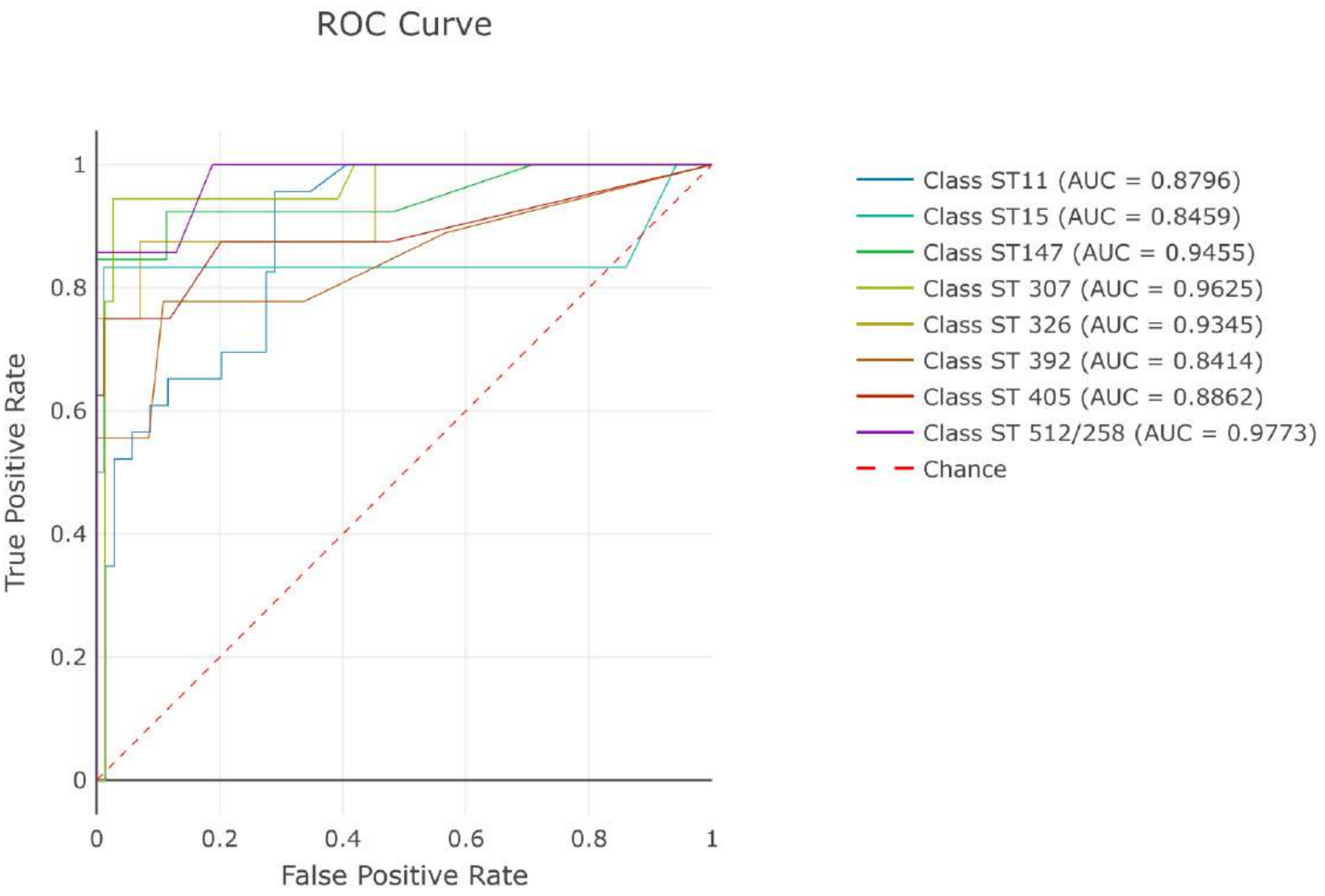

B. AUROC representation when the training was performed in the HGM and the validation in the HUCA

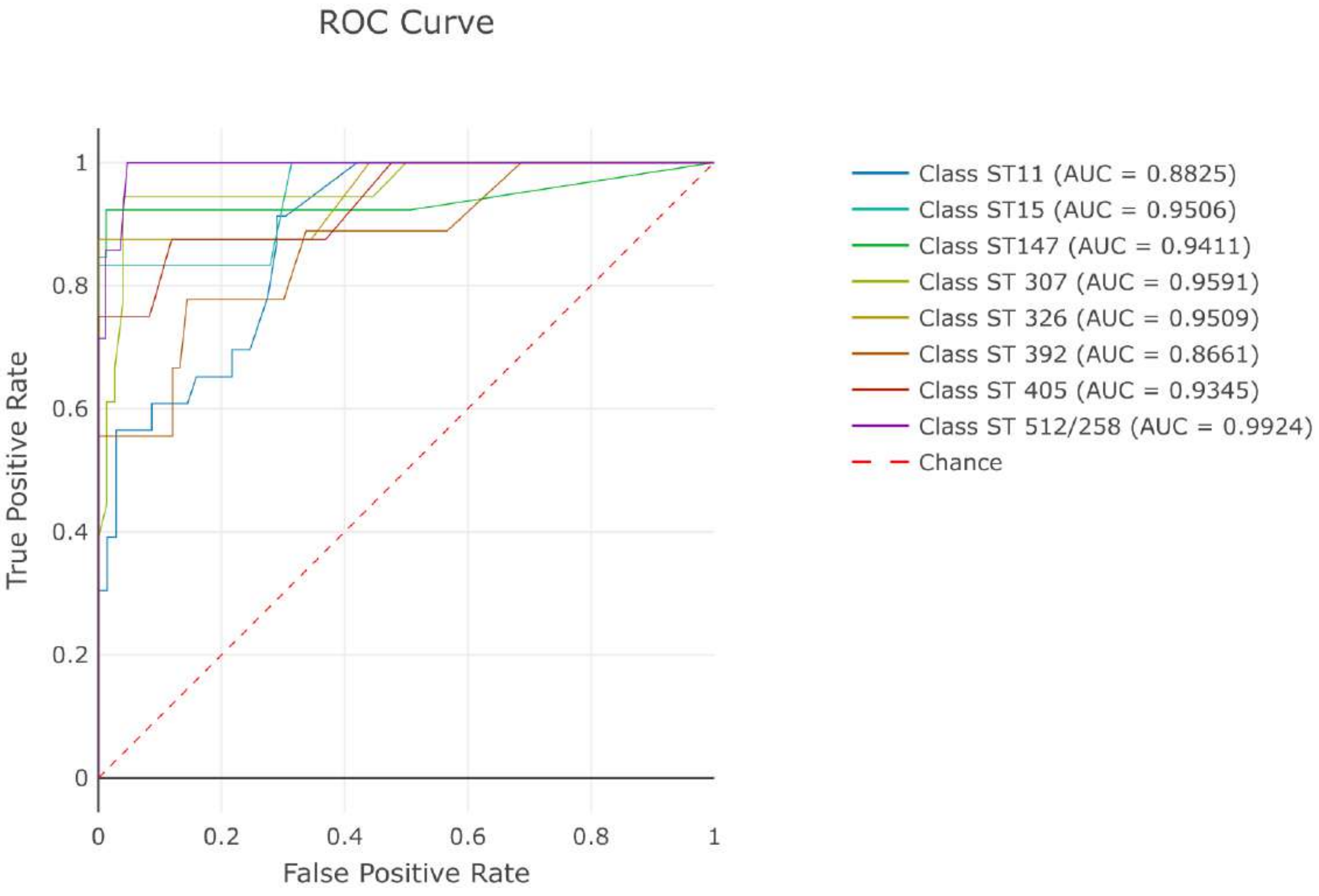

Supplement: Figure S4 — ROC curves. [file jcm.01122-24-s0004.pdf]
