# Supplementary material for: Multicenter evaluation of Fourier transform infrared (FTIR) spectroscopy as a first-line typing tool for carbapenemase-producing Klebsiella pneumoniae in clinical settings
Source: J Clin Microbiol. 2024 Nov 27;63(1):e01122-24. doi: 10.1128/jcm.01122-24 (PMC11784409; doi:10.1128/jcm.01122-24)
Supplement: Supplemental legends — Legends for Fig. S1 to S4. [file jcm.01122-24-s0005.docx]

**Supplementary material: figure leyends**

**Figure 1.** Phylogenetic tree obtained from cgSNP analysis from all carbapenemase-producing *K. pneumoniae* tested. Isolates are identified by the corresponding identification number. Each sequence type (ST) assigned is indicated with a different color in the dendogram. Besides each genomic cluster is represented with a different color on the right and identified by a latin letter.

**Figure 2**. Principal component analysis (PCA) for FTIR clustering of all carbapenemase-producing *K. pneumoniae*, using Euclidean metric distance and the average linkage method in the IR Biotyper software (Bruker Daltonik, Germay), after including the information regarding the ST of the isolate as metadata.

**Figure 3**. Principal component analysis (PCA) for FTIR clustering of all carbapenemase-producing *K. pneumoniae*, using Euclidean metric distance and the average linkage method in the IR Biotyper software (Bruker Daltonik, Germay), after including the information regarding the genomic cluster obtained from cgSNP analysis of the isolate as metadata.

**Figure 4.** AUROC representation for FTIR clustering using the RF prediction model in the Clover MSDAS software for each ST in the validation stage.
